# Microbial Poly(hydroxybutyrate-co-hydroxyvalerate) Scaffold for Periodontal Tissue Engineering

**DOI:** 10.3390/polym15040855

**Published:** 2023-02-09

**Authors:** Seubsakul Phuegyod, Sasivimon Pramual, Nungnit Wattanavichean, Supasuda Assawajaruwan, Taweechai Amornsakchai, Panithi Sukho, Jisnuson Svasti, Rudee Surarit, Nuttawee Niamsiri

**Affiliations:** 1 Department of Biotechnology, Faculty of Science, Mahidol University, Bangkok 10400, Thailand; 2 Laboratory of Biochemistry, Chulabhorn Research Institute, Bangkok 10210, Thailand; 3 School of Materials Science and Innovation, Faculty of Science, Mahidol University, Nakhon Pathom 73170, Thailand; 4 Center of Excellence for Innovation in Chemistry, Department of Chemistry, Faculty of Science, Mahidol University, Nakhon Pathom 73170, Thailand; 5 Department of Clinical Sciences and Public Health, Faculty of Veterinary Science, Mahidol University, Nakhon Pathom 73170, Thailand; 6 Department of Oral Biology, Faculty of Dentistry, Mahidol University, Bangkok 10400, Thailand; 7 Faculty of Dentistry, Siam University, Bangkok 10160, Thailand

**Keywords:** polyhydroxyalkanoates, poly(hydroxybutyrate-co-hydroxyvalerate), tissue engineering, 3D porous scaffolds, human gingival fibroblasts, periodontal ligament stem cells

## Abstract

In this study, we fabricated three dimensional (3D) porous scaffolds of poly(hydroxybutyrate-co-hydroxyvalerate) with 50% HV content. P(HB-50HV) was biosynthesized from bacteria *Cupriavidus necator* H16 and the in vitro proliferation of dental cells for tissue engineering application was evaluated. Comparisons were made with scaffolds prepared by poly(hydroxybutyrate) (PHB), poly(hydroxybutyrate-co-12%hydroxyvalerate) (P(HB-12HV)), and polycaprolactone (PCL). The water contact angle results indicated a hydrophobic character for all polymeric films. All fabricated scaffolds exhibited a high porosity of 90% with a sponge-like appearance. The P(HB-50HV) scaffolds were distinctively different in compressive modulus and was the material with the lowest stiffness among all scaffolds tested between the dry and wet conditions. The human gingival fibroblasts (HGFs) and periodontal ligament stem cells (PDLSCs) cultured onto the P(HB-50HV) scaffold adhered to the scaffold and exhibited the highest proliferation with a healthy morphology, demonstrating excellent cell compatibility with P(HB-50HV) scaffolds. These results indicate that the P(HB-50HV) scaffold could be applied as a biomaterial for periodontal tissue engineering and stem cell applications.

## 1. Introduction

Periodontitis is a chronic inflammatory oral disease caused by bacteria infection [1]. Typically, the infection destroys periodontal cells including gingival fibroblast, periodontal ligament fibroblasts, and alveolar bone, which are the supporting tissue and bone that hold the tooth. As the disease progresses, more oral tissues are damaged, causing deep pockets, which eventually lead to teeth loss if left untreated [2].

Tissue engineering has been employed to regenerate the lost periodontal tissues and restore both structure and function. In this regard, three dimensional (3D) porous scaffolds represent important components for tissue engineering as a supporting material for cell proliferation or differentiation before being applied to repair the damaged area [3,4]. Scaffolds provide attachment sites and structural guidance for cells that enable them to synthesize appropriate extracellular matrix (ECM) proteins and ultimately proliferate into functional tissues [5]. In addition, the choice of scaffold can be critical as its chemical and physical properties provide guidance cues for the cells to behave appropriately. Scaffold biomaterials for successful tooth regeneration applications should have some requirements such as being biocompatible, biodegradable, and possess mechanical properties that are consistent with the implanted area as well as being used in the appropriate amount and with an accessible volume of porosities for the diffusion of oxygen, cells, and nutrients [6,7]. To date, many polymeric materials have been reported to create biodegradable scaffolds for dental tissue engineering including poly(lactide) (PLA) [8], poly(lactide-co-glycolide) (PLGA) [9,10], and polycaprolactone (PCL) [11,12,13].

Polyhydroxyalkanoates (PHAs) are aliphatic polyesters synthesized by microorganisms to store excess carbon and energy. Poly(hydroxybutyrate-co-hydroxyvalerate) or P(HB-HV) copolymers are a member of the PHA family [14]. P(HB-HV) has shown great potential for tissue engineering with attractive characteristics of natural origin, biocompatibility, and biodegradability. The properties are adjustable by changing the content of the HV unit. P(HB-HV) are less crystalline, less stiff, and more flexible than the PHB homopolymer due to the incorporation of the HV monomer in the polymer chain [15]. Recently, studies have revealed that different types of scaffolds made with P(HB-HV) demonstrate desirable advantages for tissue engineering. The application of macroporous P(HB-8HV) matrices in the repair of full-thickness cartilage defects in rabbits in vivo was reported by Kose et al. At 8 and 20 weeks after seeding, in vivo results with chondrocyte seeded P(HB-8HV) matrices presented early cartilage formation resembling normal articular cartilage and revealed minimal foreign body reaction. This study also showed that P(HB-8HV) matrices maintained their integrity for 21 days and permitted appropriate gradual degradation and allowed for tissue remodeling to take place [16]. Abazari et al. demonstrated the increased survival rate and insulin-producing cell (IPC) differentiation potential of induced pluripotent stem cells (iPSCs) cultured on a nanofibrous 3D P(HB-5HV) scaffold in comparison with the 2D substrate. iPSCs-P(HB-5HV), as a promising cell-copolymer construct, could potentially be applied in pancreatic tissue engineering applications to diabetic patient treatment [17]. The P(HB-3HV) scaffold was tested for degradation in simulated body fluid (SBF), pH 7.4. After 8-week periods, the P(HB-3HV) scaffolds revealed about 51% weight loss along time due to the high porous structure when compared with the dense and compact films, which showed about a 9% weight loss. Culturing of MC3T3-E1 pre-osteoblast cells on the P(HB-3HV) scaffold samples obtained after 6 weeks of degradation did not lead to the formation of cytotoxic components [18]. In spite of extensive research on P(HB-HV) and their blends as scaffolds for tissue engineering, the HV molar contents of the available commercial P(HB-HV) published are 12 mol% or lower. P(HB-HV) films consisting of various HV content (5–80%) produced by *Paracoccus denitrificans* have been reported to be biocompatible with connective tissue, bone, and dermal fibroblast cells [19]. *Haloferax mediterranei* ES1 produced P(HB-HV) nanofibrous meshes were also shown to be excellent in vitro and to show in vivo biocompatibility with skin tissues [20]. As reported earlier, the more flexible P(HB-HV) with HV contents of 50 mol% can be successfully biosynthesized from bacteria *Cupriavidus necator* H16. This material has already been employed as a drug delivery platform [21,22,23]. Until now, there have been no studies available in the literature concerning the application of P(HB-50HV) produced by *C. necator* H16 as a scaffold to support cell growth and promote tissue regeneration.

In this study, the 3D porous scaffolds were fabricated from bacterial derived P(HB-50HV) via a particulate leaching method using salt particles as a strategy for the regeneration of periodontal cells. Comparisons were made with scaffold prepared from poly(hydroxybutyrate) (PHB) and poly(hydroxybutyrate-co-12%hydroxyvalerate) (P(HB-12HV)), and the well-established synthetic polycaprolactone (PCL). The scaffolds were characterized with respect to the morphology of the surface and cross section, porosity, mechanical strength, and protein absorption. Subsequently, biological performance of the scaffolds in terms of biocompatibility and cell proliferation was assessed. In this regard, human gingival fibroblasts (HGFs) [10] and periodontal ligament stem cells (PDLSCs) [24] were used since they have been widely studied for the initial evaluation of biomaterials for periodontal tissue engineering applications.

## 2. Materials and Methods

### 2.1. Materials

Poly(hydroxybutyrate) (PHB, Mw 3.5 × 10^5^ g/mol), poly(hydroxybutyrate-co-hydroxyvalerate) containing HV content 12 mol% (P(HB-12HV), Mw 2.5 × 10^5^ g/mol), polycaprolactone (PCL, Mw 6.5 × 10^4^ g/mol), and 3-(4,5-Dimethyl-2-thiazolyl)-2,5-diphenyl-2H-tetrazolium bromide (MTT) were obtained from Sigma-Aldrich (St. Louis, MO, USA). Chloroform, methanol, and ethanol were purchased from RCI Labscan (Bangkok, Thailand). Sodium chloride (NaCl) (Ajax Chemicals Ltd., Sydney, Australia) with the particle size range of 425–500 µm was obtained by sieving through an analytical sieve shaker Octagon digital (Endecotts Ltd., London, UK) using two certified sieve sizes with 425 and 500 μm. Dulbecco’s modified Eagle medium (DMEM), fetal bovine serum (FBS), and trypsin-EDTA were obtained from Invitrogen (Carlsbad, CA, USA). All chemicals and solutions were used as supplied without further purification. Poly(hydroxybutyrate-co-hydroxyvalerate) with 50% HV content (P(HB-50HV) Mw 1.69 × 10^6^ g/mol) was biosynthesized by in house bacterial cultivation according to a previously described protocol [21].

### 2.2. Characterization of Polymer Films

Thin polymeric films of PHB, P(HB-12HV), P(HB-50HV), and PCL were prepared by a casting method using 10 mL of 2% (*w*/*v*) polymer stock solution in chloroform on a clean Petri dish. Chloroform was evaporated in a fume hood at room temperature for 24 h. The final thickness of film ranged from 0.05 to 0.10 mm.

The Fourier transform infrared (FTIR) spectra of all PHA and PCL thin films were obtained with a Perkin-Elmer FTIR ATR-FTIR spectrometer (Perkin-Elmer, Spectrum GX FTIR; Shelton, CT, USA). The sample spectra were recorded over 20 scans between 400 and 4000 cm^−1^ wavenumbers at a resolution of 4 cm^−1^.

The hydrophilicity of the polymeric surfaces was examined by an optical bench-type contact angle goniometry DM-CE1 (Kyowa Interface Science, Niiza, Japan) using a sessile drop method at room temperature.

### 2.3. Fabrication and Characterization of Scaffolds

Salt-leached scaffolds of PHB, P(HB-12HV), P(HB-50HV), and PCL were fabricated following the established procedure [25]. In brief, the polymer was dissolved in chloroform to prepare a 5% (*w*/*v*) stock solution. The 1 mL polymer solution was then poured on a bed of sieved NaCl particles (with size range of 425–500 µm) in a clean glass vial. The weight ratio of porogen (NaCl) to polymer was set at 9:1. The scaffolds were placed in a fume hood at room temperature for the slow evaporation of chloroform over 2 days followed by repeated rinsing with distilled water to remove any residual salt and air-dried. All scaffolds were prepared as a cylindrical shape with 10 mm diameter and 3 mm height.

The fabricated scaffolds were mounted onto an aluminum stub, gold-coated, and then observed by scanning electron microscopy (SEM, JSM-6360; JEOL Techniques, Tokyo, Japan) with an accelerating voltage of 20 kV for the surface topography and cross section images.

The porosity or void volume fraction V_f_ (%) of the scaffold was calculated using the following equation:

V_f_ = (1 − (ρ_s_/ρ_m_)) × 100

where ρ_s_ is the apparent density of the porous scaffold and ρ_m_ is the density of the polymer material [26].

### 2.4. Compressive Mechanical Testing of Scaffolds

The scaffolds were subjected to mechanical measurements under compressive mode in order to determine the compressive stress and compressive modulus (E). The tests were performed at room temperature using a Texture analyzer (TA-XT2i, Stable Micro Systems, Ltd., Godalming, UK) with a 50 kN load cell at a crosshead speed of 0.1 mm/s [27]. Cylindrical specimens were tested under both dry and wet conditions. The load deformation curves of the samples obtained were converted into stress–strain curves. The compressive stress (MPa) was used to calculate the secant modulus according to the following equation:

Compressive modulus at 30% strain = Compressive stress (MPa)/0.3

Under the wet condition, the compressive properties of each scaffold were measured in DMEM to mimic the physiological environment [28]. The scaffolds were preconditioned by soaking in the DMEM containing 10% FBS for 24 h at 37 °C. Then, scaffolds were placed in a Petri dish containing fresh media and compressed using a similar setup as above-mentioned. Each reported value was averaged from six independent measurements.

### 2.5. Evaluation of Protein Absorption on Scaffolds

The protein absorption onto porous scaffolds was determined following a previously published protocol with some modifications [29]. The scaffold sample was cut into equal sizes (10 mm diameter and 3 mm height) and sterilized by soaking in 70% ethanol for 1 h, followed by air drying in a laminar hood. Scaffolds were incubated in 1 mL of DMEM containing 10% FBS for 24 h at 37 °C in a humid atmosphere containing 5% CO_2_. Bradford protein assays were performed to determine the residual FBS proteins left in DMEM by using bovine serum albumin (BSA) as a standard [30]. Then, 1 mL of Bradford reagent was added to 100 μL of DMEM solution and incubated for 20 min in the dark. The absorption at 595 nm was measured. The amount of FBS proteins absorbed onto the scaffold could be determined indirectly by subtracting the initial amount of proteins present in DMEM with the residual proteins left in the DMEM solution after removing the scaffold. The absorbed proteins could be reported as % (*w*/*w*) proteins absorbed per scaffold.

### 2.6. Cell Culture

The human gingival fibroblasts (HGFs) and periodontal ligament stem cells (PDLSCs) were originally obtained from American Type Culture Collection (ATCC^®^). The cells were cultured in DMEM supplemented with 10% (*v*/*v*) FBS, 100 units/mL penicillin, and 100 μg/mL streptomycin in an environment of 95% air and 5% CO_2_ at 37 °C.

### 2.7. In Vitro Cell Proliferation Study

The scaffold samples were sterilized with 70% ethanol followed by UV exposure. Each scaffold was then transferred to 48-well plates and washed with phosphate buffered saline (PBS). Prior to seeding the cells, the scaffolds were soaked with 1 mL of fresh cell culture media containing 10% FBS for 3 h at ambient temperature to precondition the scaffolds, as previously described [31]. Thereafter, the preconditioning media were removed and the cells were seeded at a density of 5 × 10^4^ cells/well. Cell cultivations on the scaffolds were carried out over 8 days for the HGF cells and 21 days for the PDLSCs cells, respectively. The cell proliferations were evaluated using the MTT colorimetric assay. For each time point, the scaffolds with cultured cells were washed twice with PBS and transferred into a new clean well. Then, 0.5 mL of MTT solution (1 mg/mL) was added to each well, followed by incubation at 37 °C for 4 h. The excess MTT solution was then removed and formazan crystals that formed in the living cells were dissolved by adding 0.5 mL isopropanol. The liquid solution measured the absorbance at 570 nm using an Epoch microplate spectrophotometer (BioTek Instruments, Inc., Winooski, VT, USA). To visualize nuclear and cytoskeletal morphologies, both the HGF and PDLSC cells were fixed with 2% paraformaldehyde and permeabilized with 0.1% Triton X-100. After washing with PBS, the nuclei were stained with Hoechst 33342 (Invitrogen Corporation, Carlsbad, CA, USA) and the actin filaments were labeled with Alexa Fluor 568 phalloidin solution (Invitrogen Corporation, Carlsbad, CA, USA). The images were collected with a confocal laser scanning microscope (FV10i-DOC; Olympus, Tokyo, Japan).

### 2.8. Statistical Analysis

Data are expressed as the mean ± SD of three independent experiments. The software package PASW Statistics 18 for Windows (SPSS Inc., Chicago, IL, USA) was used for the statistical analysis. The *p*-value < 0.05 was considered statistically significant.

## 3. Results and Discussion

### 3.1. Characterization of Polymer Films

**Figure 1:**
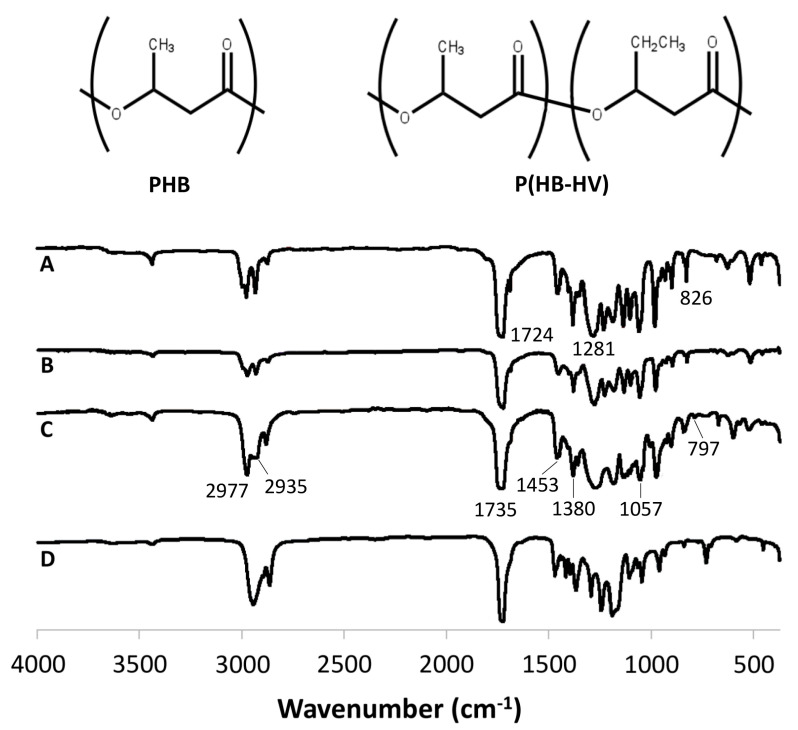
FTIR spectra of the (A) PHB, (B) P(HB-12HV), (C) P(HB-50HV) and (D) PCL polymers.

The FTIR spectra shown in Figure 1 were used to assess the functional groups present in the polymers. The FTIR spectra of the PHB, P(HB-12HV), P(HB-50HV), and PCL polymers are also shown for comparison. Since both PHAs and PCL contain ester bonds, peaks of C=O stretching were observed around 1730 and 1625 cm^−1^ for the PHAs and PCL, respectively. Both the PHA and PCL spectra also showed slightly different C–H stretching and bending, located from 3000 to 2800 cm^−1^ and from 1500 to 1000 cm^−1^ [32]. Although the PHB, P(HB-12HV), and P(HB-50HV) polymers are chemically similar, the differences in the HV composition of the polymers could be distinguished by FTIR spectra. The PHB homopolymer showed characteristic peaks at 1724 cm^−1^ for C=O stretching and 1281 cm^−1^ for C–O stretching [33,34,35]. Apart from additional peaks at 797 cm^−1^, responsible for C–H bending, the presence of HV in the P(HB-HV) copolymers could be identified by observing the FTIR peak shifts. A major shift occurred at the C=O stretching region, in which the peak shifted from 1724 cm^−1^ in PHB to 1735 cm^−1^ in P(HB-HV). The greater the change to the higher wavenumber, the higher the %HV monomer in the polymer chain. This phenomenon was also observed in other peaks such as C–O stretching at 1281 cm^−1^ and the C–H stretching region around 3000 cm^−1^. In addition, several peaks from FTIR can be used to denote the crystallinity state of different PHA polymers. The peaks at 1453, 1380, 1281, 1057, and 826 cm^−1^ shifted to a higher wavenumber when the crystallinity was low [36,37]. Our results showed that there were around five to 10 wavenumber shifts in the mentioned peaks among PHB, P(HB-12HV), and P(HB-50HV). Therefore, the PHAs used in this study were confirmed as having differences in %HV as well as their crystallinity.

The hydrophilicity of a polymer surface is the key parameter affecting cell–material interaction and the adsorption of protein on the polymer surface, which subsequently influence cell behaviors [38]. The results of the water contact angle measurements are summarized in Table 1. All samples showed contact angles of below 90° considering hydrophilic behavior. The highest contact angle value of the PCL film indicated the greater hydrophobicity of PCL than the other PHAs. The contact angle value of P(HB-50HV) was significantly higher than the other PHA films tested (*p* < 0.05). This might be due to more ethyl groups of the HV monomer present in the side chain of the copolymers [39]. Kim et al. reported a water contact angle of 79.5° of the P(HB-60HV) film produced by *Haloferax mediterranei* ES1 [20].

### 3.2. Characterization of Scaffolds

**Figure 2:**
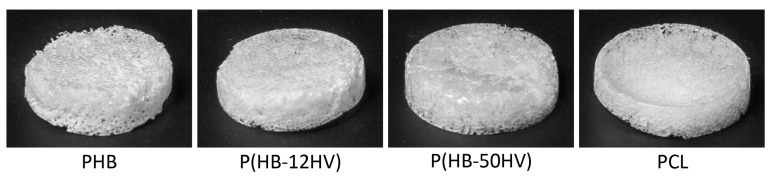
Photographic images of 3D porous scaffolds via the particulate salt leaching technique.

**Figure 3:**
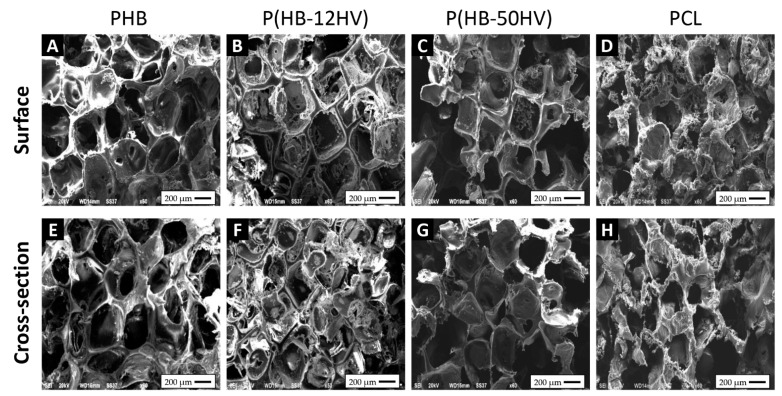
SEM micrographs of representative 3D porous scaffold samples with pore sizes ranging from 425 to 500 µm: PHB scaffold (**A**,**E**), P(HB-12HV) scaffold (**B**,**F**), P(HB-50HV) scaffold (**C**,**G**), and PCL scaffold (**D**,**H**).

The PHB, P(HB-12HV), P(HB-50HV), and PCL scaffolds were fabricated via a particulate salt leaching technique. All fabricated porous scaffolds exhibited a high porosity of 90% with a sponge-like appearance (Figure 2). The structure of the pores as well as the surface and cross-sectional topologies of the 3D porous scaffolds were examined using SEM, as shown in Figure 3. All polymeric scaffolds were similar in terms of the surface and cross-sectional topographies that comprised of interconnected open pores throughout the scaffolds. The well-tailored pore sizes ranged between 425 and 500 μm on both the surface and inside the scaffolds, suggesting sufficient surface areas for cell attachment. Furthermore, the pore shape observed was similar to the shape of the imprinted salt crystals. Our results agree with earlier findings for scaffolds with pore sizes of around 400 μm, which are considered suitable for the growth and proliferation of bone cells [40]. In general, the scaffolds were highly porous with interconnected pore networks that facilitate nutrient and oxygen diffusion and waste removal during tissue formation. The interconnected networks between open pores are also important for cellular attachment, proliferation, and migration for tissue vascularization [26,41].

### 3.3. Mechanical Properties of Scaffolds

**Figure 4:**
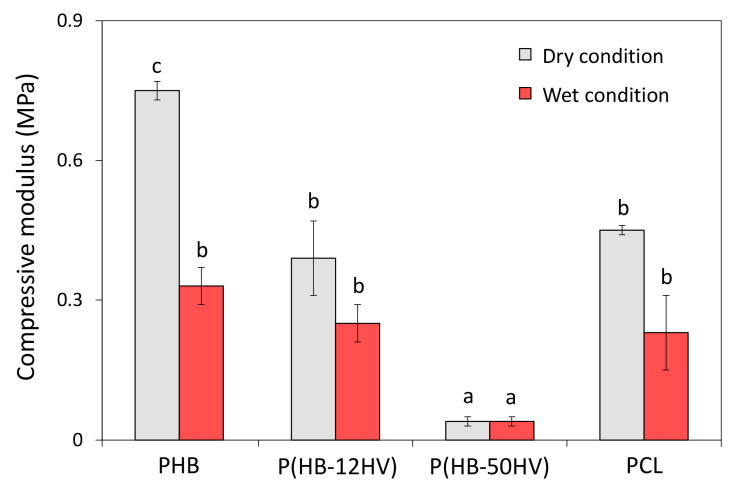
Compressive secant modulus at 30% strain compared between the dry and wet conditions. Data are reported as the average values from six independent scaffolds with standard deviations (*n* = 6). The different letters (a, b, and c) above the bar graph indicate significant differences (*p* < 0.05) between the scaffold materials tested under the same conditions.

The typical stress–strain curves obtained from the compressive stress measurement at 30% strain were used to calculate the compressive modulus of all scaffolds. As presented in Figure 4, the compressive modulus values under the dry condition of the PHB, P(HB-12HV), and P(HB-50HV) scaffolds were found to be 0.75 ± 0.02, 0.39 ± 0.08, and 0.04 ± 0.01 MPa, respectively. The compressive modulus of the PCL scaffold at 0.45 ± 0.01 MPa was not significantly different from the scaffold made with P(HB-12HV) polymers. In addition, the lowest compressive modulus was observed in the P(HB-50HV) scaffold. Our results suggest that increasing the HV content in the P(HB-HV) polymer chain at 50% could lead to a significant decrease in the compressive modulus of a 3D porous scaffold while maintaining the same % porosity. Previous studies have reported that an increase of %HV up to 50–60% could cause a lower melting temperature due to a decrease in the crystallinity of the PHA copolymers, resulting in ductile mechanical properties such as higher elongation to break and greater flexibility with a faster degradation rate under specific physiological conditions [42,43]. Among the P(HB-HV) with various %HV contents produced by *P. denitrificans*, film sheets composed of P(HB-HV) with a HV of 53–60 mol% were found to be more flexible and tougher [19]. Here, the P(HB-50HV) scaffold appeared to be the most soft and flexible scaffold in the dry state.

The wet-state mechanical properties of 3D porous scaffolds were also investigated in order to determine their compressive behavior in a realistic environment. In Figure 4, the compressive modulus values under wet conditions of the PHB, P(HB-12HV), and PCL scaffolds were found to be approximately in the same levels at 0.33 ± 0.04, 0.25 ± 0.04, and 0.23 ± 0.08 MPa, respectively, which were 62%, 36%, and 49% reduced from the dry condition, respectively. However, there was no change in the compressive modulus of the P(HB-50HV) scaffold under the wet state when compared to the dry state, which still remained at 0.04 ± 0.01 MPa. There was a clear decrease in the mechanical properties from the dry to wet state in all scaffolds, except for P(HB-50HV). Our findings are in line with earlier reports that observed decreased compressive moduli of 3D polymeric scaffolds under wet conditions, which used PBS and cell culture media [27,28]. The water molecules could intersperse and intercalate among the polymer chains that finally spread the polymer chains apart by losing the crystalline network characteristics of the polymer [44]. Notably, there was no discernable difference between the dry and wet conditions on the compressive modulus of the P(HB-50HV) scaffold. One explanation could be that the compressive modulus of P(HB-50HV) at the dry state is already quite low, and that any eventual water plasticizer effect might be too small to be detected.

### 3.4. Cell Proliferation

**Figure 5:**
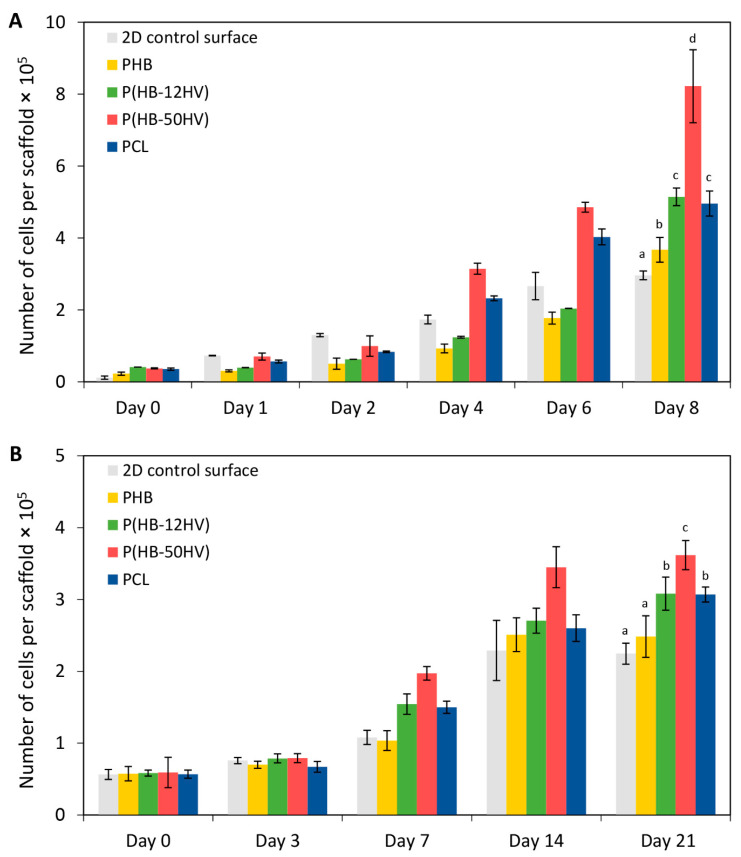
Cell proliferation of (**A**) HGF and (**B**) PDLSC cells grown on different types of 3D porous scaffolds. The values are the means with standard deviation derived from three independent scaffolds (*n* = 3). Bars labeled with different letters (a, b, c, and d) indicate significant differences within the same day (*p* < 0.05).

Early cell adhesion and proliferation are necessary in developing scaffolds for periodontal regeneration. The cell adhesion ability and proliferation enhancement of HGFs and PDLSCs on 3D porous scaffolds were studied using the MTT assay. The HGF cells were cultured on different scaffolds for 0, 1, 2, 4, 6, and 8 days. In Figure 5A, the HGF cells grew quite slowly during the first 2 days for all types of scaffolds tested. Interestingly, cell numbers at 8 days were significantly the highest for the P(HB-50HV) scaffold, which showed about a 16-fold increase from the start, followed by the P(HB-12HV) and PCL scaffolds (10-fold), PHB scaffold (8-fold), and 2D control surface (6-fold), respectively.

The proliferation ability of PDLSC cells was investigated at 0, 3, 7, 14, and 21 days, as shown in Figure 5B. Similar cell numbers were found on the 2D control surface and in all scaffolds at day 0. On the last day of the experiment, the highest number of PDLSC cells was significantly increased on the P(HB-50HV) scaffold with a 7-fold increase from the initial cell loaded compared with the P(HB-12HV) and PCL scaffolds (6-fold), and the PHB scaffold and 2D control surface (5-fold).

**Figure 6:**
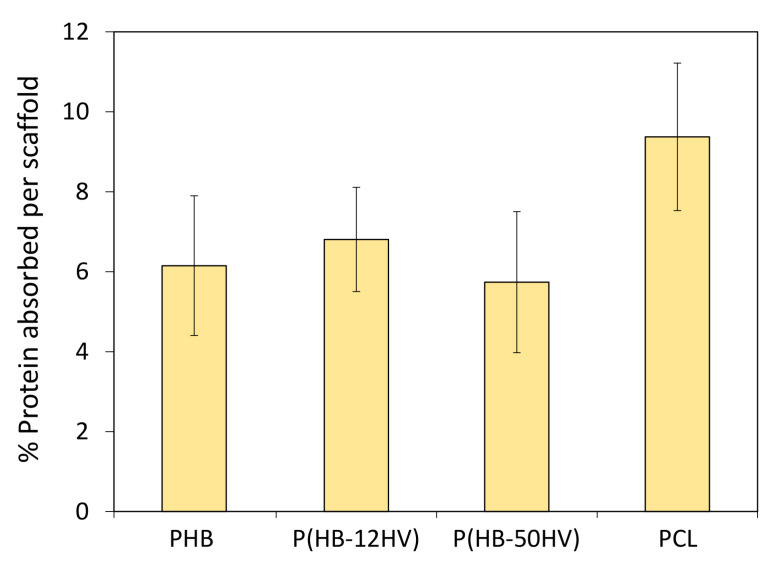
The adsorption of FBS proteins on 3D porous scaffolds. The values are the means with standard deviation derived from three independent scaffolds (*n* = 3).

In this study, it was clear that all PHA scaffolds could support the attachment and proliferation of HGF and PDLSC cells. This is the first report on the cytotoxicity and biocompatibility of P(HB-50HV) produced by *C. necator* H16 as a candidate scaffold for dental tissue engineering. However, the P(HB-50HV) scaffold appeared to be the most suitable to support both HGF and PDLSC cell growth among the three types of PHA scaffolds, and was even better than the conventional PCL scaffold. Since the chemical properties of PHA and PCL scaffolds are quite similar such as the functional group and hydrophilicity, protein absorption on the material surface is known to be one of the important considerations to promoting cell attachment and the growth of anchorage-dependent mammalian cells on a solid substratum [45]. The amount of protein absorbed on the scaffolds’ surface were found to be similar, as shown in Figure 6. Thus, the HGF and PDLSC cell proliferation was related to the mechanical properties of the 3D porous scaffolds. Many previous studies have reported that the stiffness of the material has an effect on cell attachment signaling, leading to a difference in the cell proliferation and differentiation [46,47]. These results indicate that the P(HB-50HV) scaffold had the lowest stiffness, which could promote the greatest adhesion and proliferation of HGF and PDLSC cell adhesion and proliferation, and thus should be considered as a suitable material for the tissue engineering of periodontal cells and other soft tissue-like cells. Regarding the scaffold mechanical properties, our results are in line with the finding reported previously that the fabricated PCL scaffolds with lower modulus values than the PLGA scaffolds showed a 2-fold higher growth rate of stromal cells [48].

### 3.5. Cell Morphology

**Figure 7:**
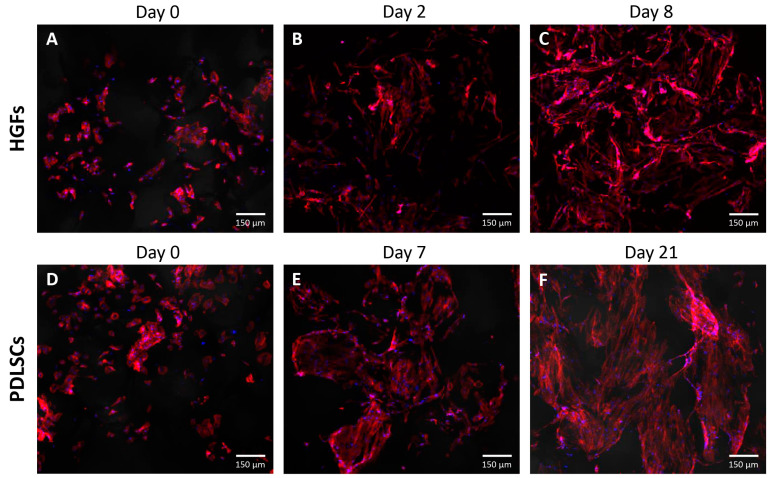
Confocal micrographs of the (**A**–**C**) HGF and (**D**–**F**) PDLSC cells cultured on the P(HB-50HV) scaffold. Cell nucleus was stained with Hoechst 33342 (blue). F-actin was stained with Alexa Fluor 568 phalloidin (red).

The morphology of healthy HGF and PDLSC cells cultured on the P(HB-50HV) scaffold was further analyzed by fluorescence staining of the nucleus and F-actin filaments. The round-shape of the HGF cells was observed at day 0 (Figure 7A) followed by cell migration and the development of interconnecting network development by day 2 (Figure 7B). After 8 days of culture, the HGF cells were distributed throughout the entire scaffold with a strong presence of F-actin, resulting in a dense interconnecting network of cells (Figure 7C).

The initial cell adhesion of PDLSC cells at day 0 were observed to be round-shape (Figure 7D). The PDLSC cells proliferated considerably well on the scaffold surface and gradually progressed to high cell density all over the cultured scaffold from day 7 (Figure 7E) to day 21 (Figure 7F). Importantly, both HGF and PDLSC cell morphology on the P(HB-50HV) scaffold showed both spindle and stellate shapes, which are typically good indicators of healthy fibroblasts [49,50]. The results showed that the biocompatibility of the P(HB-50HV) scaffold has promising potential for periodontal tissue engineering.

The scaffold made from bacterially-derived P(HB-50HV) copolymers developed in this study showed a softness property and displayed a capability to promote good proliferation and the interconnection of periodontal cells including HGF and PDLSC cells. The microbial P(HB-50HV) scaffold is not only effective for normal fibroblast cell regeneration, but also demonstrates good potential to promote stem cell proliferation. With these interesting properties, the P(HB-50HV) scaffold is an attractive material for tissue engineering strategies.

## 4. Conclusions

In this study, 3D porous scaffolds made from PHB, P(HB-12HV), P(HB-50HV), and PCL polymers were successfully fabricated via the salt leaching method with similar properties in terms of chemical functionality, surface hydrophilicity, surface topography, % porosity, and serum protein absorption. Furthermore, the P(HB-50HV) scaffolds were distinctively different in their compressive modulus by having the lowest stiffness among all of the scaffolds tested. The proliferation of dental cells including HGF and PDLSC cells was investigated with four different types of scaffolds. Interestingly, the P(HB-50HV) scaffold showed the highest proliferation of both HGF and PDLSC cells over all of the PHA scaffolds and the control PCL scaffold. Cells grown on the P(HB-50HV) scaffold had the characteristic of healthy fibroblasts in forming highly dense interconnecting networks. Taken together with the hydrophilicity, softness property, greater cell proliferation, and morphology of dental cells grown on the P(HB-50HV) scaffold, these results confirm the possibility of using a microbial-derived P(HB-50HV) scaffold as a biomaterial for periodontal tissue engineering and stem cell applications.

## Figures and Tables

**Table 1:** Surface hydrophilicity of the polymer films.

Type of Polymers	Water Contact Angle (°)
PHB	70.2 ± 3.5 ^a^
P(HB-12HV)	67.9 ± 2.1 ^a^
P(HB-50HV)	76.8 ± 1.8 ^b^
PCL	81.4 ± 1.8 ^c^

(Mean ± SD, *n* = 3, different superscript letters indicate a significant difference at *p* < 0.05).

## Data Availability

The data presented in this study are available on request from the corresponding author.
